# Testing the excitability of human motoneurons

**DOI:** 10.3389/fnhum.2013.00152

**Published:** 2013-04-24

**Authors:** Chris J. McNeil, Jane E. Butler, Janet L. Taylor, Simon C. Gandevia

**Affiliations:** ^1^Neuroscience Research AustraliaRandwick, NSW, Australia; ^2^School of Health and Exercise Sciences, University of British ColumbiaKelowna, BC, Canada; ^3^Faculty of Medicine, University of New South WalesSydney, NSW, Australia

**Keywords:** motoneuron, H-reflex, F-wave, tendon jerk, V-wave, CMEP, MEP

## Abstract

The responsiveness of the human central nervous system can change profoundly with exercise, injury, disuse, or disease. Changes occur at both cortical and spinal levels but in most cases excitability of the motoneuron pool must be assessed to localize accurately the site of adaptation. Hence, it is critical to understand, and employ correctly, the methods to test motoneuron excitability in humans. Several techniques exist and each has its advantages and disadvantages. This review examines the most common techniques that use evoked compound muscle action potentials to test the excitability of the motoneuron pool and describes the merits and limitations of each. The techniques discussed are the H-reflex, F-wave, tendon jerk, V-wave, cervicomedullary motor evoked potential (CMEP), and motor evoked potential (MEP). A number of limitations with these techniques are presented.

## Introduction

The motoneuron was described as the “final common path” of the nervous system (Sherrington, 1906) and has been a focal point of neuroscience research for over a century. Since the introduction of the terminology, the designation of “final common path” has been frequently expanded to include not only the α-motoneuron but also the muscle fibers which it innervates (i.e., the motor unit) (e.g., Denslow and Hassett, 1942). This expanded definition is sensible from a functional perspective because movement requires contraction of muscle fibers and the properties of muscle fibers are largely dictated by the properties of the motoneuron which innervates them (see Burke, 1981; Henneman and Mendell, 1981; Binder et al., 1996 for reviews).

Motor unit properties have been directly studied in animals (e.g., Burke et al., 1971; Peter et al., 1972) and are directly related to the size of the motoneuron. In brief, motoneurons with a large soma (and hence a large axonal diameter) have a low input resistance, high firing threshold, brief after-hyperpolarization, fast conduction velocity, and their muscle fibers have a fast twitch contraction time, high twitch tension and a poor resistance to fatigue. As the size of motoneurons decreases, these responses gradually shift to the opposite ends of their spectra. Output properties of human motoneurons can be gleaned from whole-nerve stimulation, spike-triggered averaging (e.g., Milner-Brown et al., 1973; Thomas et al., 1990) or intraneural stimulation (e.g., Thomas et al., 1990). Measurement of somato-dendritic properties is more difficult and frequently relies on reflex and antidromic inputs to the motoneurons.

Like the assessment of motor unit properties, tests of motoneuron pool excitability in humans are necessarily indirect. Before we discuss the most common methods used to test excitability of the human motoneuron pool, it is first necessary to define our use of the term “excitability” in this review. For our purposes, the term “excitability” is a relative one. That is, if the same input is delivered to the motoneuron pool before and after an intervention (e.g., muscle fatigue), do more or fewer motoneurons generate action potentials after the intervention? A bigger or smaller output would represent a net increase or decrease in motoneuron excitability, respectively. The change in excitability will reflect the balance of inhibition and facilitation but it is difficult to determine the mechanism in a given situation; e.g., a decrease in excitability could be due to either an increase in inhibition or a decrease in facilitation. Further, any change in “excitability” may not apply uniformly across the whole motoneuron pool.

Regardless of the methodology or the intervention studied (e.g., acute vs. chronic), the goal of testing human motoneuron excitability is the same: to know more about the status of the motoneuron pool. The aim of this review is to discuss briefly the existing methodologies which test motoneuron excitability by evoking compound muscle action potentials with particular focus on the benefits and limitations of each. Discussion of the H-reflex, F-wave, tendon jerk, V-wave, cervicomedullary motor evoked potential (CMEP) and motor evoked potential (MEP) is included below and summarized in Table 1. There are a number of single motor unit approaches with their own advantages and disadvantages but these methods are largely beyond the scope of this review. However, it deserves emphasis that these approaches provide useful information but usually only about a limited number of motoneurons which have a low-threshold in a voluntary contraction.

**Table 1:** **Brief summary of the methodologies used to test excitability of motoneurons in humans**.

**Response**	**Key information**	**Advantages/recommendations**	**Disadvantages/caveats**
H-reflex	Method: submaximal stimulation of a peripheral motor nerve. Volley: single in group I muscle afferents (and other afferents). Potential: motoneurons activated by Ia excitation. Note: motoneurons recruited according to size principle.	Potentially painless. Possible to test in relaxation or weak contraction.	Not entirely monosynaptic. Limited to soleus and a few other motoneuron pools in relaxation. Test conditions must be painstakingly maintained. Subject to presynaptic inhibition. Subject to post-activation (homosynaptic) depression. Affected by changes in axonal excitability.
F-wave	Method: supramaximal stimulation of a peripheral motor nerve. Volley: single antidromic in motor axons. Potential: motoneurons activated by antidromic excitation. Note: only a small number of motoneurons backfire; in muscles with an H-reflex at rest, the response occurs preferentially in large motoneurons because the antidromic volley collides with the H-reflex impulse in small motoneurons.	A direct method which does not rely on afferents. Interpretation is clearest when tested in relaxation.	Relatively insensitive to motoneuron excitability. Necessary to average or measure many responses. Limited to distal muscles. Can be painful. Contaminated by H-reflex when recorded during weak contraction.
Tendon jerk	Method: tendon taps with a reflex hammer. Volley: multiple from muscle spindle primary endings (and other afferents). Potential: motoneurons activated by Ia excitation.	Painless. Simple to administer. May be the only test available for a muscle. Tested in relaxation.	Not entirely monosynaptic. Difficult to replicate mechanics of tendon tap across trials and conditions. Thixotropic state of intrafusal fibers needs to be controlled.
V-wave	Method: supramaximal stimulation of a peripheral motor nerve during a voluntary contraction. Volley: single in both muscle afferents and motor axons. Potential: motoneurons activated by Ia excitation. Note: only motoneurons whose axons are first cleared by collision of descending volitional and antidromic impulses contribute.	Best for strong (maximal) contractions.	Difficult to identify which elements of the motor system are responsible for any change seen in the response. Will vary with motoneuron firing rate. Can be painful.
CMEP	Method: submaximal stimulation at the level of the pyramidal decussation. Volley: single descending in the corticospinal tract. Potential: motoneurons activated by corticospinal excitation. Note: onset latency must be monitored to avoid root stimulation.	Not subject to conventional presynaptic inhibition. Largely monosynaptic. Unnecessary to average many responses. Possible to test in relaxation and contraction.	Painful. Not entirely monosynaptic. Difficult to obtain in some subjects. Difficult to obtain in some motoneuron pools.
MEP	Method: submaximal transcranial magnetic stimulation of the motor cortex. Volley: multiple descending in the corticospinal tract. Potential: motoneurons activated by corticospinal excitation. Note: some motoneurons can discharge more than once.	Painless. Large proportion of the motoneuron pool can be accessed under appropriate conditions. Possible to test in relaxation and contraction.	Affected by both cortical and spinal excitability and hence cannot measure either in isolation. Not entirely monosynaptic. Although not painful, can be unsettling at high stimulus intensities.

*Note: Submaximal/supramaximal refers to the stimulus strength relative to the current required to evoke the maximal compound muscle action potential (M_max_)*.

## H-reflex

**Figure 1:**
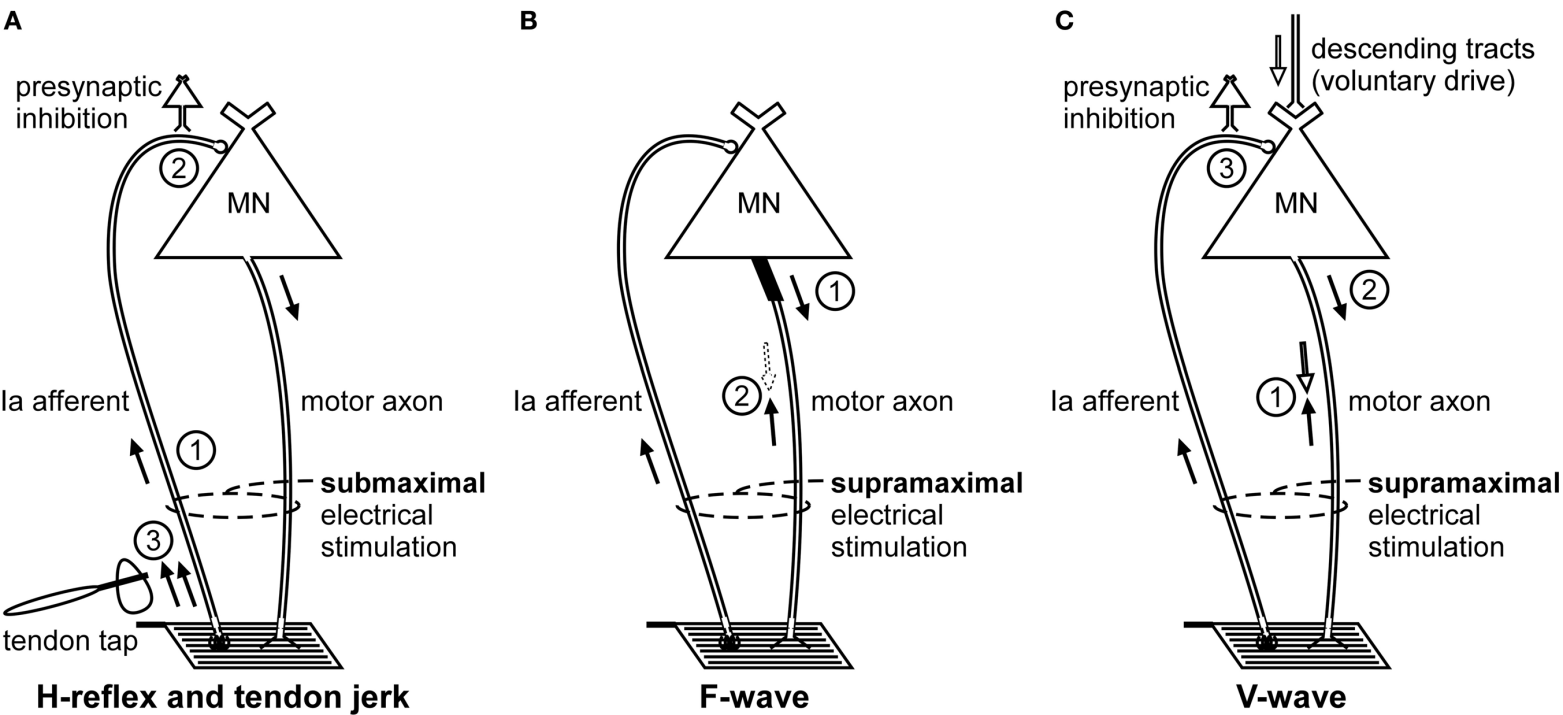
**Schematic representation of the volleys and pathways involved in the production of the H-reflex, tendon jerk, F-wave, and V-wave.** Only the most critical elements are labeled so see text for a complete description of the factors which can influence each response. **(A)**, #1—electrical stimulus evokes a single afferent volley which recruits motoneurons for the H-reflex according to the size principle; #2—presynaptic inhibition can influence afferent input to the motoneuron; #3—tendon tap evokes multiple volleys which arrive at the motoneuron over 25 ms. **(B)**, #1—a small number of motoneurons may discharge to produce F-waves after antidromic impulses reach their soma; #2—at rest, F-waves are likely to be limited to large motoneurons due to reflex activation of smaller motoneurons and collision with the antidromic volley prior to the soma. **(C)**, #1—in motor axons conducting orthodromic impulses of voluntary drive, voluntary and antidromic impulses will collide; #2—reflex response which travels along motor axons cleared by the collision described in point #1 will contribute to the V-wave; #3—presynaptic inhibition can influence afferent input to the motoneuron.

Stimulation of a peripheral nerve can evoke a reflex response termed the Hoffmann or H-reflex (Magladery and McDougal, 1950) because it was first described in the soleus muscle by Hoffmann (1918). The H-reflex reflects the response of the motoneuron pool to a volley from large-diameter primary muscle spindle afferents (Figure 1A). Most commonly recorded as a multi-unit response from surface electromyographic activity, it is possible to record an H-reflex in single motor units (e.g., Trontelj, 1968; Ashby and Zilm, 1982; Burke et al., 1984; Miles et al., 1989). Similar to the descending input during voluntary contractions, the synaptic Ia input will recruit motoneurons in an orderly fashion from smallest to largest (slow to fast motor units; Buchthal and Schmalbruch, 1970) according to the Henneman size principle (e.g., Henneman and Mendell, 1981). Delivery of a series of progressively stronger stimuli generates a recruitment curve of the H-reflex and the muscle compound action potential (M-wave). In brief, H-reflex size increases with stimulus intensity until it reaches a maximum. This point can occur when further increases in intensity do not result in further increases in the net excitatory input to the motoneurons. Alternatively, a maximum can be reached because further increases in intensity reduce H-reflex size due to collision of afferent-evoked orthodromic impulses with antidromic impulses evoked in the motor axons that contribute to the growing M-wave. The H-reflex recorded at the tipping point is referred to as *H*_max_. Comparison of H-reflex size to the maximal M-wave enables an estimate of the motoneuron pool involved in the H-reflex. For the soleus muscle in most subjects the percentage of involved motoneurons is ~50% (Taborikova and Sax, 1968). A recruitment curve provides additional parameters (e.g., H-reflex threshold, slope of the ascending limb of the recruitment curve) which give insight into H-reflex input/output relationship (e.g., Zehr, 2002; Klimstra and Zehr, 2008). Finally, data about recruitment curves improve the validity of comparisons of data across time or experimental conditions.

Initially believed to be a purely monosynaptic Ia reflex (Hoffmann, 1922; Magladery et al., 1951; Paillard, 1955), it has since been established that the relatively long rise time of the compound excitatory postsynaptic potential (EPSP) (1.9–2.1 ms in soleus motoneurons; Birnbaum and Ashby, 1982; Burke et al., 1983) enables disynaptic (and possibly oligosynaptic) Ia pathways and Ib afferents to exert an influence on the H-reflex (Burke et al., 1983, 1984). Because the threshold to electrical stimulation and the conduction velocity of the fastest Ia and Ib afferents are probably not greatly different, both afferents feature prominently in the initial volley which arrives at the spinal cord (Pierrot-Deseilligny et al., 1981). Ib afferents acting via an inhibitory interneuron do not prevent the monosynaptic Ia EPSPs which initiate the H-reflex but do terminate the EPSPs with inhibitory postsynaptic potentials (IPSPs) at an interval as brief as 1 ms (Pierrot-Deseilligny et al., 1981). The first experimental, rather than theoretical, evidence of this non-reciprocal group I inhibition was a disynaptic limitation of the size of the quadriceps H-reflex (Marchand-Pauvert et al., 2002). Although this inhibition limits the size of the H-reflex (Burke et al., 1984), the majority of the response recorded under most conditions reflects monosynaptic Ia afferent input to the motoneuron pool. The influence of disynaptic or oligosynaptic input on motoneuron recruitment is determined by the size of the compound monosynaptic EPSP relative to the recruitment threshold of each motoneuron. That is, early recruited units in the response will be recruited by monosynaptic input but the discharge of the last recruited motoneurons will reflect the balance between monosynaptic excitation and dior oligosynaptic inhibition and/or excitation. Hence, a change in H-reflex size occurs primarily through a change in this balance. This is true regardless of the size of the test H-reflex.

For more than 50 years, the H-reflex has been widely used as a test of the excitability of the human motoneuron pool. However, there are a number of caveats for this test which are commonly ignored or under-appreciated, despite a wealth of experimental data (e.g., Paillard, 1955) and detailed discussion of the technique (e.g., Schieppati, 1987; Pierrot-Deseilligny and Mazevet, 2000; Zehr, 2002; Pierrot-Deseilligny and Burke, 2005). A major mechanism that has long been known to alter the size of the H-reflex is the degree of presynaptic inhibition of Ia terminals (e.g., Frank and Fuortes, 1957; Eccles et al., 1961; Hultborn et al., 1987; see Rudomin and Schmidt, 1999 for review). Some other key mechanisms which can influence the size of the H-reflex include post-activation depression or homosynaptic depression (e.g., Magladery and McDougal, 1950; Crone and Nielsen, 1989; see Hultborn and Nielsen, 1998 for review) and contributions of oligosynaptic pathways (e.g., Pierrot-Deseilligny et al., 1981; Burke et al., 1983, 1984). Finally, in testing the H-reflex, it is difficult to be sure that the afferent volley itself is constant because activity leads to axonal hyperpolarization and reduced excitability in sensory and motor axons (e.g., Kiernan et al., 1997; Vagg et al., 1998) such that the same stimulus intensity is likely to activate fewer afferent axons after a voluntary contraction. See Pierrot-Deseilligny and Burke (2005) for a detailed discussion of these and other mechanisms.

The size of an H-reflex is sensitive to changes in subject posture (Hugon, 1973) and attention (Bathien and Morin, 1972), so it is critical that these factors vary as little as possible when collecting H-reflexes. Further, these factors make day-to-day comparisons of the H-reflex particularly difficult. The H-reflex is also strongly influenced by the frequency of stimulation as post-activation depression reduces the size of a second response elicited within 10 s of the first. To avoid this reflex attenuation, the stimulation frequency of repeated H-reflexes would ideally not exceed 0.1 Hz. However, collection of the requisite large number of responses at this rate is time-consuming and so a faster stimulation rate is more practical even if some post-activation depression remains. As the decay of post-activation depression is curvilinear (Magladery and McDougal, 1950), stimulating at 0.2–0.3 Hz strikes an acceptable balance between the level of depression and the time required to collect the responses (Pierrot-Deseilligny and Mazevet, 2000). The frequency of stimulation can be increased as high as 4 Hz during voluntary contraction because the post-activation depression seen in relaxed muscle is greatly attenuated or abolished (Burke et al., 1989; see also Stein et al., 2007) possibly because the extra impulse evoked by the electrical stimulus will have negligible impact on transmitter release from Ia afferents which are already discharging (Stein and Thompson, 2006). Although the size of the effect was relatively small, another factor to consider is the regularity of the stimuli. An interstimulus interval which varied between 0.5 and 1.5 s (mean of 1 s) evoked a larger H-reflex compared to stimulation at a constant interval of 1 s (Hoehler et al., 1981).

A practical limitation of H-reflexes is that they can only be obtained consistently from a small number of muscles during relaxation (typically soleus, flexor carpi radialis, quadriceps). However, this list of muscles expands greatly if stimulation is given while the subject performs a weak voluntary contraction. This ensures some motoneurons are discharging repetitively, brings others closer to threshold and thus increases motoneuron excitability. Burke and colleagues (1989) identified a number of other benefits to testing the H-reflex during voluntary contraction which include: the abolition of post-activation depression (homosynaptic depression); larger response sizes with lower stimulus intensities and hence a clearer distinction between the end of the M-wave and the onset of the H-reflex; a focus of the reflex response to the active motoneuron pool so that specific reflex arcs can be studied. Additional benefits are a reduction in the levels of homonymous (Fournier et al., 1983) and heteronymous Ib inhibition (Pierrot-Deseilligny and Fournier, 1986). Presynaptic inhibition is reported to be the same at rest and during steady contraction (Meunier and Pierrot-Deseilligny, 1989; Nielsen and Kagamihara, 1993) and so appears to represent neither an advantage nor a disadvantage to testing the H-reflex during voluntary contraction.

Thus, far, this section has described homonymous connections, that is from the stimulated Ia afferents to the motoneuron pool of the same muscle. However, there are also heteronymous links between Ia afferents of one nerve to the motoneuron pools of muscles supplied by a different nerve. For example, stimulation of the femoral nerve delivers monosynaptic Ia excitation not only to the quadriceps motoneurons but also to the soleus motoneuron pool (e.g., Bergmans et al., 1978; Meunier et al., 1990). Hence, appropriately-timed stimulation of the femoral nerve facilitates the soleus H-reflex. Heteronymous Ia monosynaptic excitation has been demonstrated in both the upper (e.g., Cavallari and Katz, 1989; Marchand-Pauvert et al., 2000) and lower limbs (e.g., Meunier et al., 1993) of humans. The utility of these connections is the ability to test the excitability of a motoneuron pool without the serious effect of activity-dependent changes in the excitability of the homonymous afferents.

## F-wave

Although not termed the F-wave until 1950 (Magladery and McDougal, 1950), this late response to stimulation of a peripheral nerve was first described by Eccles and Pritchard (1937). Described as a recurrent discharge (e.g., Eccles, 1955), the F-wave reflects backfiring of a small number of motoneurons which are reactivated by antidromic impulses following supramaximal stimulation of a peripheral nerve (Figure 1B). Because F-waves are small (often less than 0.5 mV) and inconsistent in both size and shape, large numbers of responses are collected for averaging (Lin and Floeter, 2004). The variability of onset latency and morphology would cause considerable phase cancellation if raw F-waves were averaged online so potentials must be measured individually or full-wave rectified prior to averaging (Espiritu et al., 2003). In a clinical setting, rather than calculating the average response to a large number of stimuli, persistence (the percentage of stimuli evoking a response) and the difference in latency between the onset of the slowest and fastest single motor unit potentials are used as objective measures of properties of the motoneuron pool.

The production of an F-wave by a given motoneuron is believed to depend on the excitability of the axon initial segment (Eccles, 1955), and perhaps also the first node of Ranvier (Gogan et al., 1984) The passage of the antidromic impulse to the soma will make these sites transiently refractory. If the axon initial segment remains refractory when the antidromic impulse evokes a somato-dendritic action potential, an orthodromic action potential will not be initiated in the axon to be propagated to the periphery and recorded as an F-wave. Intraneural stimulation of single thenar motor axons indicates that generation of an F-wave in an individual motoneuron is probabilistic and occurs rarely (after <2% of stimuli). Further, their incidence is unrelated to motor axon conduction velocity or twitch force (Thomas et al., 2002). This suggests a contribution from a mixed population of motoneurons. However, in more common experimental and clinical testing, the stimulus intensity is higher and the motoneurons which generate F-waves are likely to be limited to large motoneurons due to reflex activation of smaller motoneurons and collision with the antidromic volley prior to the soma (Espiritu et al., 2003).

Interpretation of the F-wave is simplest when the muscle is relaxed at the time of stimulation but responses can be recorded during voluntary contraction (e.g., Giesebrecht et al., 2011). The disadvantage to collecting responses during contraction is that a collision between voluntary orthodromic impulses with antidromic impulses will leave some motor axons clear to transmit an H-reflex to the muscle and thereby obscure the F-wave. This problem is exacerbated as the strength of contraction increases because a greater proportion of motor axons will see their antidromic impulse obliterated before it reaches the soma. During strong voluntary contractions, few antidromic impulses will reach the soma because of collision with the orthodromic voluntary and reflex action potentials. In these circumstances, any recorded potential would almost certainly be a V-wave (see V-wave section which follows).

It has been suggested that F-waves are a useful and direct measure of motoneuron excitability (e.g., Fisher, 1992) but subsequent reports suggest that F-waves only offer a flawed measurement of motoneuron excitability (Hultborn and Nielsen, 1995; Espiritu et al., 2003; Lin and Floeter, 2004; Pierrot-Deseilligny and Burke, 2005). Chief among the limitations is the relative insensitivity of F-waves to changes in motoneuron excitability. Nonetheless, they are potently depressed following fatiguing voluntary contractions (Khan et al., 2012; Rossi et al., 2012). A reduction in F-waves can be caused not only by inhibition of the motoneuron pool but by facilitation as well (Eccles, 1955; Hultborn and Nielsen, 1995). In a strongly facilitated motoneuron pool, the antidromic impulse which invades the soma will be followed by a somato-dendritic action potential at such a short interval that the axon initial segment is still refractory (Eccles, 1955). However, this may not be a practical limitation in human studies based on results with voluntary contractions (Giesebrecht et al., 2011). The other key criticisms of the F-wave concern the practice of comparing H-reflexes and F-waves in an effort to separate events at the level of the motoneurons; that is, to gain insight into changes in presynaptic inhibition versus changes in motoneuron excitability (e.g., Leis et al., 1995). Hultborn and Nielsen (1995) questioned the validity of such a comparison on three theoretical bases. The first relates to the collision between antidromic impulses and H-reflex discharges in slowly conducting motor axons. An enhancement of motoneuron excitability (e.g., by voluntary contraction) will increase the size of the H-reflex and thereby actually decrease the number of motoneurons capable of producing an F-wave because of a greater number of collisions. Second, the motor unit populations involved in the two potentials differ. The H-reflex involves small motor units with slowly conducting axons whereas, for reasons described above, the F-wave is likely to involve large motor units with fast axons. The third point relates to the mode of activation of the two responses (afferent vs. antidromic). To illustrate their concerns, Hultborn and Nielsen (1995) conducted a simple experiment which showed that F-waves could be an order of magnitude less sensitive than H-reflexes to changes in motoneuron excitability (although both responses were facilitated by a conditioning stimulus to the femoral nerve).

## Tendon jerk

For limb muscles in which an H-reflex is not easily obtained, an alternative method of testing motoneuron excitability is a tendon jerk reflex. A tendon tap with a reflex hammer or more controlled means will create a stretch-induced barrage of discharges from muscle spindle primary endings and other afferents (Figure 1A). The size of the muscle spindle afferent volley depends on the mechanics of the tap and the sensitivity of the sensory endings. This sensitivity can be changed by contraction of the intrafusal muscle fibers on which the endings are located. Like the electrically-induced H-reflex, the mechanically-induced tendon jerk cannot be considered purely monosynaptic (e.g., Burke et al., 1983). However, unlike the relatively synchronous volley of the H-reflex, the afferent volley of the tendon jerk includes multiple discharges of a single Ia afferent and lasts for 25 ms (Burke et al., 1983, 1984). As a result, the afferent volley which arrives at the motoneurons is more dispersed for the tendon jerk than the H-reflex. This is one of several differences between the two reflexes which invalidate a comparison of the two responses as a surrogate measure of efferent drive to the muscle spindles (i.e., fusimotor activity) (Burke et al., 1983).

While ongoing fusimotor drive must potentially alter the size of the afferent volley evoked by a tendon tap, the history of prior fusimotor drive exerts a potent effect on the volley (Polus et al., 1991)due to the “thixotropic” behavior of the intrafusal muscle fibers (see Proske et al., 1993 for review). If a muscle is undisturbed or lengthened slowly following fusimotor activation, intrafusal fibers remain taut as actin-myosin cross bridges are maintained or re-established in a more stretched position. Conversely, passive shortening after fusimotor activation will maintain the cross bridges but cause the intrafusal fibers to become slack (Polus et al., 1991). This can produce large changes in background spindle firing rates. When spindles are held taut as a result of prior activity, the response to a tendon tap is increased. Note that this will have the opposite effect on the H-reflex as the increased background firing will lead to increased presynaptic inhibition (Polus et al., 1991).

The long held view that background fusimotor drive is necessary to elicit a tendon jerk from relaxed muscle is false (Burke et al., 1981). The relative ease of obtaining a tendon jerk in one muscle compared to another is likely to depend on intrinsic spinal mechanisms. While the tendon jerk has the advantage of being simple to administer, changes in its size depend on many factors, beginning with the mechanical status of the tendon and transmission of the transient lengthening to the receptors.

## V-wave

First described by Upton and colleagues (1971), the volitional wave (V-wave) is a variation of the H-reflex which is recorded during a voluntary contraction. In contrast to the submaximal stimulation used to evoke an H-reflex (see H-reflex section), a V-wave is evoked by supramaximal stimulation of a peripheral mixed nerve (Figure 1C). The supramaximal stimulus generates antidromic impulses in all motor axons as well as impulses in all group Ia afferents provided the stimulus intensity is ≥4× motor threshold (Gracies et al., 1994). In motor axons involved in the voluntary contraction, there is a collision between voluntary orthodromic and the evoked antidromic impulses which leaves these axons clear to transmit a reflex response to the muscle. Conversely, motor axons not involved in the contraction, do not contribute to the production of the V-wave because any reflex response will either collide with the antidromic impulse or the antidromic impulse will reach the soma first and leave these motoneurons refractory when the afferent volley arrives.

The V-wave is influenced by many factors (e.g., strength of voluntary contraction and the range of maximal firing rates within a motoneuron pool) and the consequent difficulty in interpreting a change to the response raises questions about its usefulness as an independent measure of motoneuron excitability. During a maximal voluntary contraction, the size of the V-wave is proposed to indicate the level of descending voluntary drive conveyed by the motoneurons (Aagaard et al., 2002). According to this proposal, an increase in V-wave size indicates increased motoneuron discharge rates or recruitment (Aagaard et al., 2002) which may reflect increased supraspinal input to the motoneuron pool. However, it is important to recall that motoneuron discharge rate reflects not only supraspinal input to the motoneuron but the response to all inputs which arrive at the motoneuron so the origin of an increase in V-wave size is uncertain. The possible sensitivity of the V-wave to supraspinal input is unlike that of the H-reflex which is largely dependent on events at a spinal level (see H-reflex section). This distinction has led to the recent application whereby changes in the V-wave are compared to those in the H-reflex before and after training to distinguish between supraspinal and spinal neural adaptations (Aagaard et al., 2002; Vila-Cha et al., 2012).

## CMEP

**Figure 2:**
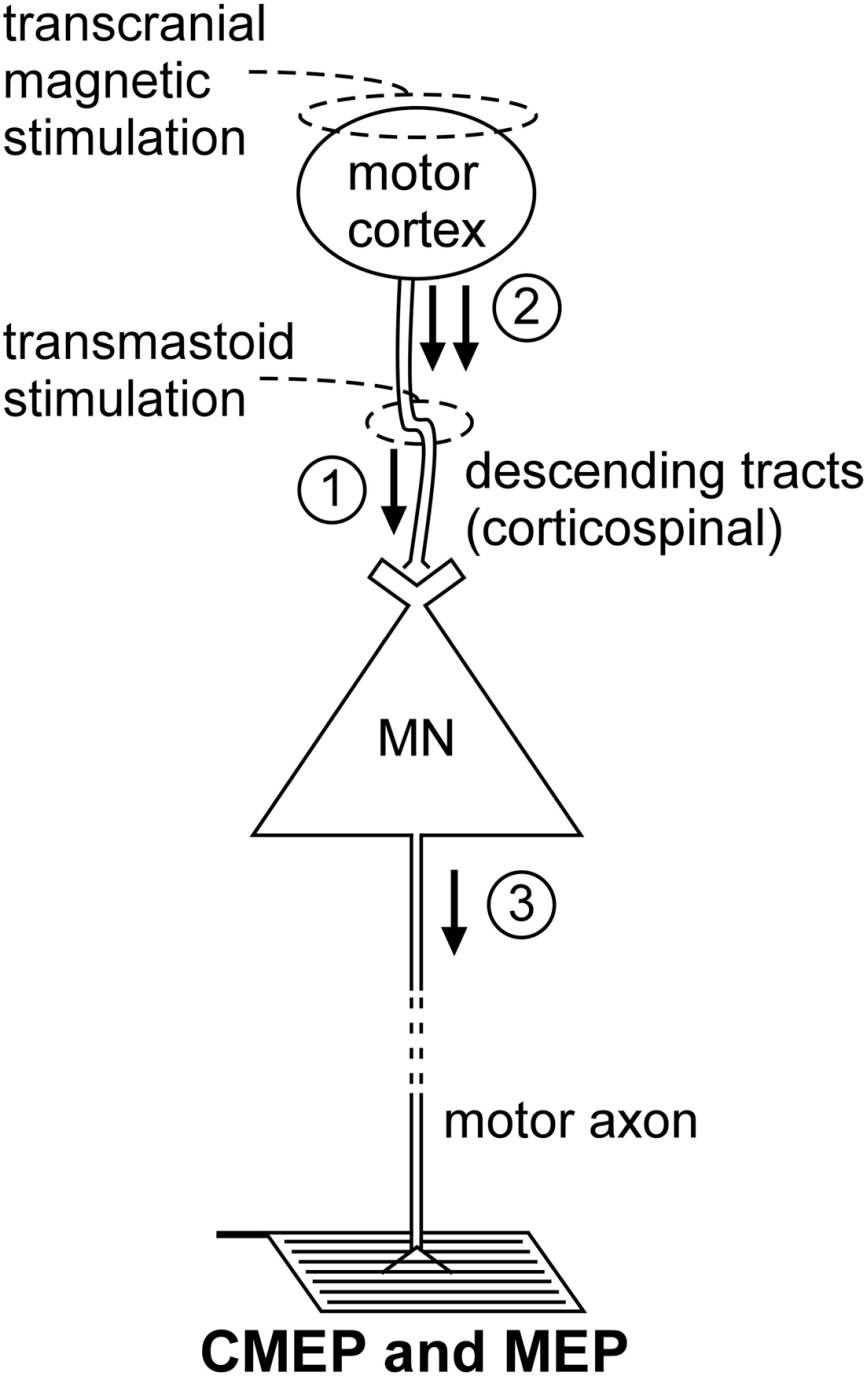
**Schematic representation of the volleys and pathways involved in the production of the CMEP and MEP.** Only the most critical elements are labeled so see text for a complete description of the factors which can influence each response. #1—transmastoid stimulation evokes a single volley which is not subject to conventional presynaptic inhibition; #2—transcranial magnetic stimulation evokes multiple descending volleys;#3—transcranial magnetic stimulation can cause multiple discharges of a single motoneuron so that MEP size can exceed that of the maximal compound muscle action potential (*M*_max_).

Non-invasive electrical or magnetic stimulation of spinal tracts can evoke large, short-latency responses in arm and leg muscles (Ugawa et al., 1991, 1994; Gandevia et al., 1999; Martin et al., 2008; see Taylor and Gandevia, 2004 for review). To evoke responses in arm muscles, axons are activated at the level of the cervicomedullary junction near the pyramidal decussation (Figure 2). Consequently, such a response is generally termed a CMEP. Electrical stimulation is accomplished by passing a brief high-voltage pulse between electrodes fixed near the mastoid processes. For magnetic stimulation, a double-cone coil is placed at the back of the head, with the center of the coil near the inion. Several terms describe these forms of stimulation and they are often used interchangeably. These include cervicomedullary, transmastoid, brainstem, or corticospinal tract stimulation. However, because the stimulus site can vary, not all the terms are actually synonymous. For example, responses with corticospinal components can be obtained in leg muscles with electrical stimulation over the cervical or thoracic spine (Martin et al., 2008). Responses produced by stimulation over the thoracic spine, are referred to as thoracic spine MEPs (TMEPs; Martin et al., 2008) rather than CMEPs.

Descending motor pathways other than the corticospinal tract (as well as ascending pathways) will be activated by the stimulus, but there is strong evidence that the CMEP is primarily the result of motoneuron activation by a single descending volley elicited by excitation of corticospinal axons (Berardelli et al., 1991; Ugawa et al., 1991; Gandevia et al., 1999; Taylor et al., 2002). The singular nature of the descending volley was confirmed by epidural recording in anaesthetized patients (Rothwell et al., 1994). In awake subjects, a collision experiment in which an ulnar nerve stimulus given before the brainstem stimulus (Berardelli et al., 1991) caused complete occlusion of the CMEP in abductor digiti minimi. In contrast, an ulnar nerve stimulus did not fully occlude the response to transcranial electric (Day et al., 1987) or magnetic stimulation (Hess et al., 1987) of the motor cortex which induce multiple corticofugal volleys and multiple discharges from some motoneurons. Additional collision experiments demonstrate that the CMEP is largely conducted via the large-diameter axons of the corticospinal tract. Brainstem stimulation given at appropriate times relative to electrical (Ugawa et al., 1991) or magnetic stimulation of the motor cortex (Berardelli et al., 1991; Gandevia et al., 1999; Taylor et al., 2002) largely occluded the motor cortical evoked potential (MEP) which suggests that the two stimuli activate many of the same corticospinal axons.

There are two important attributes of CMEPs which make this stimulation technique the most direct method to test motoneuron excitability to synaptic input in conscious humans (Martin et al., 2008). First, there is evidence that they have a large monosynaptic component in the upper limb (Petersen et al., 2002) and probably in the lower limb (Martin et al., 2008). Second, the descending tracts are not subject to conventional presynaptic inhibition due to primary afferent depolarization (Nielsen and Petersen, 1994; Jackson et al., 2006). This latter feature is in contrast to the H-reflex pathway (see Rudomin and Schmidt, 1999 for review). However, CMEPs must also be influenced by non-monosynaptic inputs although these have not been identified, and changes in CMEPs after strong voluntary contractions are postulated to reflect some presynaptic mechanism other than conventional presynaptic inhibition (Gandevia et al., 1999). Another advantage of the CMEP is that large responses can be evoked and hence averaging of large numbers of responses is not usually necessary.

Despite the advantages of corticospinal stimulation, like all stimulation techniques, it has limitations. A practical disadvantage is the discomfort produced by the stimulus. Most subjects will tolerate the stimuli but some find them prohibitively painful. The issue of pain is particularly relevant for stimuli delivered while the subject is relaxed because the discomfort is much less during muscle contraction and decreases as the level of voluntary effort increases. Apart from the issue of greater transient discomfort for the subject, the pain of stimulation when the subject is relaxed can indirectly affect the data. The size of the CMEP is sensitive to motoneuron excitability and so data collected in relaxation can be contaminated by weak inadvertent contraction if the subject instinctively “tenses up” in anticipation of the stimulus.

Another disadvantage of corticospinal stimulation is the inability or difficulty in obtaining responses of sufficient size in some subjects and some motoneuron pools. Even in subjects who tolerate stimuli within the normal range of intensities, it may not be possible to activate motoneurons via descending tract stimulation and record a valid CMEP. This occurs when the stimulus intensity required to evoke a response also activates nerve roots distal to the motoneuron soma. Such direct activation of the motor axon will mean that the “CMEP” is contaminated by a direct motor response and may not reflect motoneuron excitability accurately. The presence of nerve root stimulation can be identified in two ways: an abrupt ~1–2 ms reduction in onset latency of the CMEP with an increase in stimulus intensity; or the absence of a large increase in CMEP size (relative to the CMEP recorded in relaxation) if a given stimulus is delivered during a weak voluntary contraction.

Corticospinal (transmastoid) stimulation has recently been paired with transcranial magnetic stimulation (TMS) as a novel means to test motoneuron responsiveness during ongoing muscle activity and fatigue without the confounding influence of unknown levels of descending voluntary drive (McNeil et al., 2009, 2011a,b,c). With this technique, a corticospinal stimulus is delivered 100 ms after a strong conditioning TMS pulse which transiently (~200 ms) silences descending drive. The interruption of descending drive briefly stops motoneuron output so the excitability of motoneurons can be tested in a state of artificial relaxation (McNeil et al., 2009) without stopping the task and thereby altering the progression of fatigue.

## MEP

TMS of the motor cortex (Figure 2) is widely used to test “motor cortical” excitability but is included here because of the profound impact of motoneuron excitability on the size of the MEP. This effect is best demonstrated by the comparison of MEPs recorded from a muscle during relaxation and voluntary contraction. Regardless of stimulus intensity (e.g., Di Lazzaro et al., 1998; McNeil et al., 2011a), MEP size increases markedly from relaxation to a weak contraction and the principal mechanism for this shift is enhanced motoneuron excitability (Hess et al., 1987; Taylor et al., 1997; Di Lazzaro et al., 1998). Hence, researchers must exercise caution when interpreting changes in MEP size as changes in “cortical” excitability. To make this claim, a valid test of motoneuron excitability must be performed to eliminate the possibility that the change in MEP size is mediated at a motoneuronal level. Even then, changes in MEP size may not represent changes in “cortical” excitability as there are significant non-monosynaptic contributions to MEPs so that changes at premotoneuronal sites can modify MEP size. For example, for muscles of the upper limb other than intrinsic hand muscles, significant excitation occurs through the C3–4 propriospinal system (Gracies et al., 1991).

## Conclusions

One element of testing motoneuron excitability which needs further investigation is the matter of specificity. That is, if an increase or decrease in excitability is noted with one method (e.g., CMEP), is the same change evident when other methods (e.g., H-reflex) are applied? We recently compared the effects of fatigue on CMEPs (TMEPs) and F-waves (Giesebrecht et al., 2011) and there are the previously described comparisons between H-reflexes and F-waves (Hultborn and Nielsen, 1995) and H-reflexes and V-waves (Aagaard et al., 2002; Vila-Cha et al., 2012). However, additional comparisons of this nature would increase our understanding of the mechanisms involved and provide insight into the validity of different methods under various conditions.

### Conflict of interest statement

The authors declare that the research was conducted in the absence of any commercial or financial relationships that could be construed as a potential conflict of interest.
